# Expression of transcriptional factor EB (TFEB) in differentiating spermatogonia potentially promotes cell migration in mouse seminiferous epithelium

**DOI:** 10.1186/s12958-018-0427-x

**Published:** 2018-10-25

**Authors:** Yue Liu, Yanqin Hu, Li Wang, Chen Xu

**Affiliations:** 1 Department of Histology, Embryology, Genetics and Developmental Biology, Shanghai Jiao Tong University School of Medicine, Shanghai, 200025 China; 2 Shanghai Key Laboratory for Reproductive Medicine, Shanghai, 200025 China

**Keywords:** Spermatogenesis, TFEB, Spermatogonia, Cell migration

## Abstract

**Background:**

Spermatogenesis is a complex process involving the self-renewal and differentiation of spermatogonia into mature spermatids in the seminiferous tubules. During spermatogenesis, germ cells migrate from the basement membrane to cross the blood-testis barrier (BTB) and finally reach the luminal side of the seminiferous epithelium. However, the mechanism for regulating the migration of germ cells remains unclear. In this study, we focused on the expression and function of transcriptional factor EB (TFEB), a master regulator of lysosomal biogenesis, autophagy and endocytosis, in spermatogenesis.

**Methods:**

The expression pattern of the TFEB in mouse testes were investigated by Western blotting and immunohistochemistry analyses. Either undifferentiated spermatogonia or differentiating spermatogonia were isolated from testes using magnetic-activated cell sorting based on specific cell surface markers. Differentiation of spermatogonia was induced with 100 nM retinoic acid (RA). shRNA was used to knock down TFEB in cells. TFEB expression was detected by immunofluorescence, qRT-PCR, and Western blotting. Cell migration was determined by both transwell migration assay and wound healing assay applied to a cell line of immortalized spermatogonia, GC-1 cells.

**Results:**

During testicular development, TFEB expression was rapidly increased in the testes at the period of 7 days post-partum (dpp) to 14 dpp, whereas in adult testis, it was predominantly localized in the nucleus of spermatogonia at stages VI to VIII of the seminiferous epithelial cycle. Accordingly, TFEB was observed to be mainly expressed in differentiating spermatogonia and was activated for nuclear translocation by RA treatment. Moreover, knockdown of TFEB expression by RNAi did not affect spermatogonial differentiation, but significantly reduced cell migration in GC-1 cells.

**Conclusion:**

These findings imply that regionally distinct expression and activation of TFEB was strongly associated with RA signaling, and therefore may promote cell migration across the BTB and transport along the seminiferous epithelium.

**Electronic supplementary material:**

The online version of this article (10.1186/s12958-018-0427-x) contains supplementary material, which is available to authorized users.

## Background

Spermatogenesis is a complex process that takes place in the testis and depends on the cooperation between somatic Sertoli cells and developing germ cells across the seminiferous epithelium. In the seminiferous epithelium, Sertoli cells and germ cells are arranged in a highly polarized fashion to efficiently support spermatogenesis by establishing an adequate environment for the production of sperm as well as providing a means of transporting the developing germ cells [1]. During spermatogenesis, the type A spermatogonia undergo mitotic cell divisions to generate type A1 to A4 cells, and some of the type A4 cells differentiate into intermediate spermatogonia, which then divide once to form type B spermatogonia. Then, tetraploid primary spermatocytes are generated from the type B cells and subsequently undergo meiosis to produce diploid secondary spermatocytes and haploid spermatids [2, 3]. Any alteration of these events, which are tightly regulated and precisely coordinated, perturbs spermatogenesis and may lead to male infertility.

In coordination with these cell divisions, the various types of germ cells migrate from the basement membrane to the adluminal side of the seminiferous tubules. One of the key migration steps is when the preleptotene spermatocytes (preL), differentiated from type B spermatogonia, migrate across the blood-testis barrier (BTB), specifically at stage VIII of the seminiferous epithelial cycle [4, 5]. This migration occurs concomitantly with a peak in retinoic acid (RA) levels present at stages VII and VIII of the seminiferous epithelium cycles, and directs the sequential and distinct programs of spermatogonial differentiation and meiotic initiation [6, 7]. The capacity of RA to control spermatogonial differentiation, meiotic initiation as well as maintenance of the cycle of the seminiferous epithelium has been well studied over the last couple of years, whereas the relationship between RA and cell migration associated with meiotic initiation is less well understood.

Cell migration is a highly coordinated, multi-step process under the tight control of multiple signaling events. Several studies have shown that the microtubule and F-actin based cytoskeletons in Sertoli cells are crucial for the transport of germ cells into the seminiferous epithelium [4, 8, 9]. However, the molecular mechanisms by which germ cells themselves regulate the timely and efficient migration across the BTB remains virtually unknown. Recently, intracellular vesicular trafficking related to either endocytosis or autophagy, coordinated by the endosome, autophagosome and lysosome systems, has been described as regulators of cell migration [10–15]. Numerous autophagy mediated cargoes and endosomal factors have been shown to modulate cell migration, and the pathways that regulate adhesion turnover or endocytic trafficking have emerged as major players in the transport and recycling of the matrix proteins, adhesion proteins and chemotactic receptors involved in the local regulation of each step of cell migration [11, 12, 16].

A gene network regulating lysosomal biogenesis and function was discovered recently, at the center of which was the master gene regulator, transcription factor EB (TFEB). TFEB recognizes and binds E-box sequences (5’-CANNTG-3′) present in most of the lysosomal related genes [17, 18]. Moreover, TFEB was also found to modulate the expression of genes involved in processes such as lysosomal biogenesis, lysosomal acidification, exocytosis, autophagy and endocytosis [19–21]. Activation and nuclear translocation of TFEB is mainly controlled by its phosphorylation status, which is modulated in response to various stimuli, such as growth factors and nutrients, and depends on the effects of two major players: the mechanistic target of rapamycin complex 1 (mTORC1) and the phosphatase, calcineurin [22–27]. Very recently, enhancement of TFEB expression and activity has been shown in several cancer cells where it helps to increase tumor growth and malignancy through enhancing cancer cell autophagy and upregulating the endosomal-lysosomal compartment [28, 29]. However, the role and regulation of TFEB in spermatogenetic cells in testis, including spermatogonia, has not been investigated.

To address this question, we investigated the expression pattern of TFEB in mouse testis and spermatogonia and used knockdown experiments to test the role of TFEB in migration and differentiation. In the present study, we show for the first time that TFEB is expressed in differentiating spermatogonia and is activated in response to RA signaling, and therefore potentially plays a role in promoting of cell migration.

## Methods

### Animals

C57BL/6 mice were purchased from the Shanghai Laboratory Animal Center. All of the mice were acclimated in the Animal Center of Shanghai Jiao Tong University School of Medicine. Animal experiments were conducted according to the International Guiding Principles for Biomedical Research Involving Animal, as promulgated by the Society for the Study of Reproduction. This research program was approved by the ethic committee of Shanghai Jiao Tong University School of Medicine (NO. A2015–034).

### Histology and immunohistochemistry (IHC)

Testes were fixed using Bouin’s solution and were embedded in paraffin. Specimens were sliced into 5 μm thick sections and mounted on glass slides, followed by deparaffinization and rehydration. The sectioned testicular and epididymal tissues were then stained with hematoxylin and eosin (H&E) and observed under a microscope (Olympus BX53, Tokyo, Japan).

For IHC staining, paraffin sections were dewaxed and rehydrated, followed by antigen retrieval through boiling the tissue for 15 min in 10 mM citrate buffer, pH 6.0. Then, the Histostain LAB-SA Detection kits (Invitrogen, MD, USA) were applied according to the manufacturer’s instructions. Primary antibody against TFEB (1:100 dilution, Santa Cruz Biotechnology, CA, USA) and the control applied normal IgG were performed overnight at 4 °C. The sections were stained using DAB and nuclei were counterstained with hematoxylin. Digital images were taken under a microscope (Olympus BX53).

### Magnetic-activated cell sorting

Magnetic-activated cell sorting was used to isolated spermatogonia from testes based on cell specific surface markers modified from a previously described method [30]. Briefly, testicular cells were collected from 7-day-old C57BL/6 mouse testes by enzymatic digestion with 1 mg/ml collagenase IV (Gibico, USA), 2.5 mg/ml hyaluronidase (Sigma, USA) and 1 mg/ml DNase I (Sigma), and followed by differential plating in DMEM/F12 medium supplemented with 10% fetal bovine serum (FBS) (Gibico) for 2 h at 34 °C to remove potential contamination of Sertoli and myoid cells. Then, the single cell suspensions were incubated with rat anti-mouse CD90.2 antibody (anti-Thy1, 1: 20 dilution; Miltenyi Biotec, Germany) or rat anti-mouse CD117 antibody (anti-cKit, 1: 20 dilution; Miltenyi Biotec) in 0.1 ml of PBS containing 1% FBS at 4 °C for 20 min. The cells were further sorted on an MS separation column (Miltenyi Biotec) according to the manufacturer’s instructions and separated into positive and negative fractions (Additional file 1: Figure S1).

### Flow cytometry assay

Detection of cell purity was carried out by indirect immunofluorescent staining in conjunction with flow cytometry. The immunofluorescent staining and flow cytometry assay were performed as described [31]. The isolated spermatogonia were fixed in 4% paraformaldehyde for 10 min at room temperature. Cell samples were labeled with GFRA1 monoclonal antibody (1:200 dilution; Santa Cruz Biotechnology), c-Kit polyclonal antibodies (1:200 dilution; Abcam, USA) and subsequently with fluorescence-labeled secondary antibodies (1:500 dilution; Biotium, USA). Flow cytometry measured the spermatogonia fluorescence signals (Becton Dickinson, Beckman Coulter, Brea, CA, USA). Cell Quest software analyzed the emission originating from at least 10,000 to 20,000 events (Beckman Coulter).

### Sertoli cell and germ cell preparation

Sertoli cell isolation was carried out as described [32, 33] with some modifications. Briefly, testes of adult mice were decapsulated in HBSS containing collagenase (1 mg/ml) for 20 min and seminiferous tubules were collected by sedimentation. Then, seminiferous tubules were dispersed in a HBSS solution containing collagenase (1 mg/ml)/hyaluronidase (1 mg/ml)/DNase (0.4 mg/ml) for 20 min at 34 °C. After washing with PBS, an additional digestion step was performed with accutase cell dissociation reagent (Innovative Cell Technologies, USA) for 15 min at 34 °C. The tubular pellet was washed with PBS and Sertoli cells were freed from the seminiferous epithelium by resuspending the pellet in DMEM/F12 medium containing 5% FBS. Myoid cells were removed by differential adhesion at the first 30 min and Sertoli cells were further cultured overnight at 34 °C. The main germ cell fraction in the supernatant was pelleted by centrifugation at 300 g. During Sertoli cell culture, contaminating germ cells were removed by hypotonic shock.

### Analyses of gene expression

Total RNA was isolated using Trizol (Invitrogen) and first-strand cDNA was synthesized using PrimeScript RT Master Mix (Takara, DaLian, China) for reverse transcription-polymerase chain reaction (RT-PCR). For real-time PCR, SYBR Green PCR Master Mix (Takara) were used in accordance with the manufacturer’s protocol (Applied Biosystems, USA). Transcript levels were normalized relative to those of β-actin. PCR conditions were 95 °C for 5 min, followed by 40 cycles at 95 °C for 15 s, and 60 °C for 43 s. Each PCR was run at least in triplicate. Primers used for PCR are referred to a previous study [34] and listed in Additional file 1: Table S1.

### Western blotting analysis

Mice testes or cultured cells were homogenized in RIPA lysis buffer (Thermo Fisher Scientific, USA) containing protease inhibitor cocktail (Roche, USA) on ice for 30 min. Then centrifuged at 12000 g, 10 min, 4 °C. The proteins in the supernatant were collected and the protein concentrations were determined by the BCA Protein Assay Kit (Thermo Fisher Scientific).

Protein samples (20 μg) were separated by using 8–16% denaturing polyacrylamide gels, then transferred to polyvinylidene difluoride (PVDF) membranes (Millipore, USA) by using a semi-dry transfer apparatus (Bio-Rad, USA). Membranes were blocked with 5% bovine serum albumin (BSA) for 1 h at room temperature and immunoblotting was performed overnight at 4 °C with the TFEB antibodies (1:5000 dilution; Santa cruz) or β-actin antibodies (1:4000 dilution; Cell Signaling Technology, USA), followed by incubation with secondary antibody conjugated to HRP (Jackson ImmunoResearch, USA). Signals were generated by enhanced chemiluminescence (Millipore) and detected by luminescent image analyzer (GE imagination LAS 4000, USA).

### Immunofluorescence analysis

Cell slides or smears were prepared and then fixed with 4% paraformaldehyde for 20 min at 4 °C. Nonspecific binding sites were blocked with 10% BSA/PBS for 60 min at room temperature, followed with 0.1% TritonX-100 permeable treatment for 10 min. Sections were incubated with the TFEB antibodies (1:200 dilution; Santa cruz), GFRA1 antibody (1:200 dilution; Santa cruz) overnight at 4 °C. Then, fluorescence-labeled secondary antibodies (donkey anti–rabbit Alexa Fluor 488, donkey anti–mouse Alexa Fluor 555, 1:500 dilution; Jackson ImmunoResearch) were used. Nuclei were counterstained with DAPI (Sigma-Aldrich). The fluorescence signals were detected under a laser scanning confocal microscope (Carl Zeiss LSM-510, Germany) equipped with an argon laser (488 nm), a He/Ne laser (543 nm), an EC Plan-NEOFLUAR 63×/1.25 objective and a LD LCI Plan-APOCHROMAT 25×/0.8 objective (Zeiss). Digital images were taken and processed using Aim software (Zeiss Systems).

### Primary cell culture of spermatogonia

Thy1 positive spermatogonia were seeded in 6-cm dishes coated with laminin (BD Biosciences, USA) at a concentration of 5000 cells/well in α-MEM medium (Gibico) supplemented with 1% FBS, 1× non-essential amino acids (NEAA) (Gibico), 1× N2–1 (Gibico), 4 ng/ml glial cell-derived neurotrophic factor (GDNF) (ProteinTech, USA) and 1000 U/ml LIF (ProteinTech) [30, 35]. All cultures were maintained at 34 °C in a humidified 5% CO_2_ incubator. In RA inducing differentiation experiment, 100 nM all-trans RA (Sigma) was added to the culture medium for the indicated periods of time to allow spermatogonial differentiation.

### RNA interference by lentivirus transduction

The TFEB shRNA targeting to 5′-GCAGCAGGCTGTCATGCATTA-3′ was produced and cloned into the pLenti X1 Puro lentiviral vector (pLenti X1 Puro-shRNA-eGFP-1), which contained an enhanced green fluorescence protein (EGFP) as a reporter gene. Meanwhile, control shRNA lentiviral vector was also constructed. Production of the packaging recombinant viruses were performed by Cyagen (Guangzhou, China). In lentivirus transduction, isolated spermatogonia or GC-1 cells were cultured in six-well plates at a density of 2 × 10^5^ cells per well. Recombinant viruses in culture medium containing 8 μg/mL of polybrene with a muiltiplicity of infection (MOI) of 5 were used for transduction of cells in each well. After infection, the plates were incubated at 34 °C and the medium was changed 24 h later. The transduction efficiency was determined by EGFP expression with flow cytometry analysis at 48 h to 72 h after transduction. The knock down of TFEB expression was determined using the quantitative PCR method.

### Transwell assay

Cells were cultured with DMEM culture medium containing 10% fetal bovine serum (FBS) for 24 h for routine digestion. Cell concentration was adjusted to 1 × 10^5^/ml using serum free DMEM culture medium. A cell suspension (100 μl) was added to the upper chamber of the transwell plate. The lower chamber of the transwell plate was innoculated with 500 μl of DMEM culture medium containing 10% FBS. Then the tranwell plate was cultured in an incubator at 37 °C with 5% CO_2_. About 24 h later, the plate was taken out and cotton swab was used to wipe out the cells in the transwell room, followed by PBS wash for three times. Then the transwell was soaked in 95% ethanol solution for 20 min to fixation and in crystal violet solution for 5 min, followed by PBS wash for three times. The penetrated cells were counted under a microscope. Each group was set with five transwell rooms, and six view fields were observed for each transwell room. Average value was calculated and obtained. Experiments were conducted for three times to obtain the average value.

### Wound healing assay

The wound healing assay was performed according to a previously described method [36]. Briefly, 4 × 10^5^ cells were cultured in a 6-well plate until they reached a near confluent monolayer. This monolayer was subsequently scratched with a 100 μl culture tip. Cellular migration from the two wound fronts was tracked and migration distances recorded after 24 h using a live imaging system (Nikon, Japan). At least five imaging views were investigated on each plate to quantify the migration rates.

### Statistical analysis

All data were analyzed using SAS 8.2 software, and results are presented as mean ± SD. Group comparisons were made using Student’s t-test where appropriate. One-way analysis of variance (ANOVA) test was used assuming a two-tail hypothesis with *P* < 0.05. Differences were considered statistically different when *P* < 0.05.

## Results

### Expression and localization of TFEB in testis

**Fig. 1:**
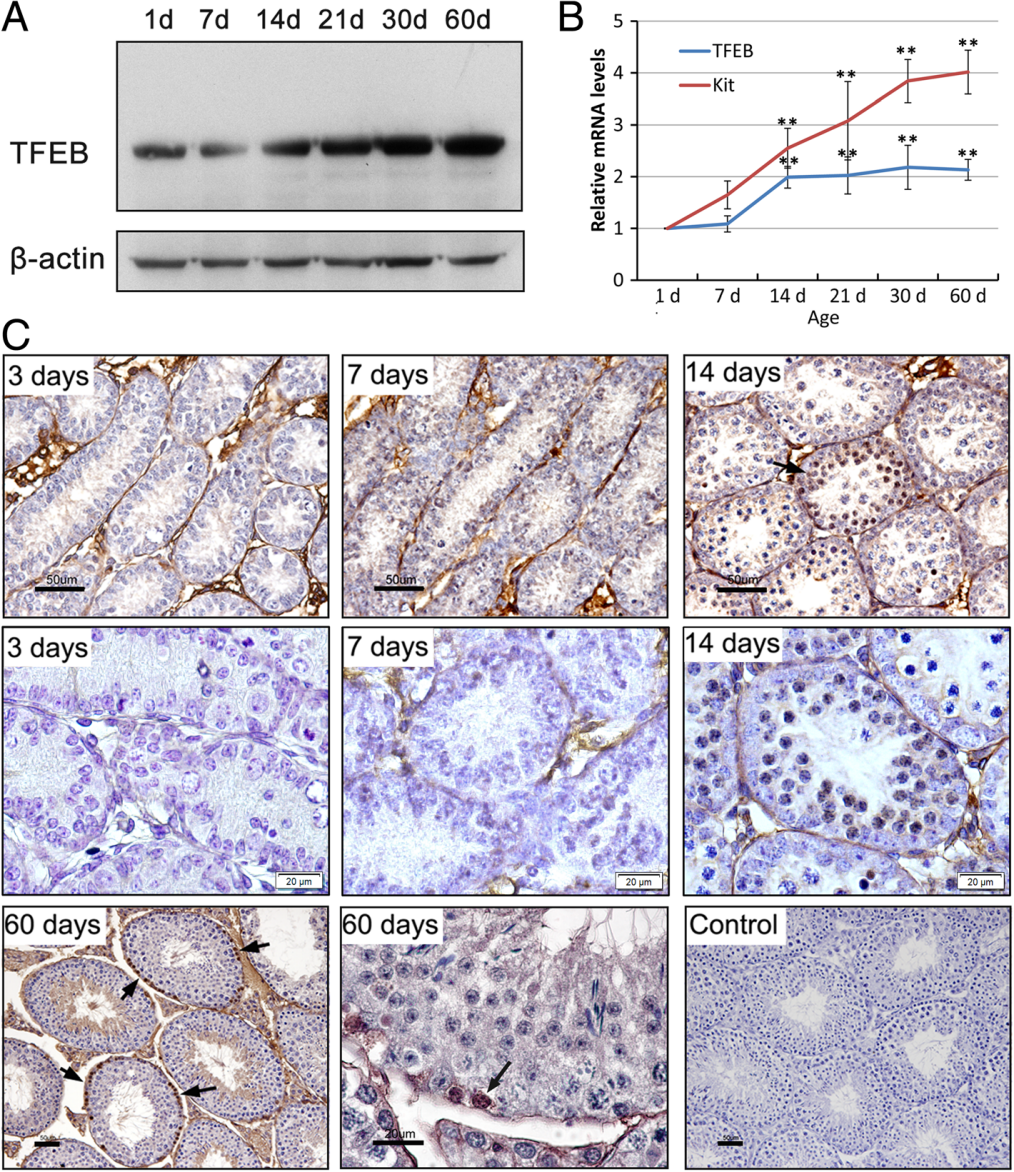
Expression of TFEB in mouse testes during development. **a-b** Western blot and quantitative PCR detection of TFEB in 1-, 7-, 14-, 21-, 30- and 60-day-old testes, showed a rapid increase in TFEB levels between 7- and 14-day-old testes. Quantitative PCR detection of Kit to confirm the spermatogenetic cell development. Error bars represent SD (*n* = 5). **p < 0.01. **c** Immunohistochemical staining of TFEB in 3-, 7-, 14- and 60-day-old testes sections, showed TFEB expression was little stained in 3- and 7-day testes and significantly positive stained in 14- and 60-day testes. Arrows indicate the staining of TFEB in nucleus. Bar: 50 μm or 20 μm

We used immunohistochemistry and immunoblotting to investigate the expression pattern of TFEB in murine testis and seminiferous epithelium. Immunoblotting analysis indicated that TFEB levels increased during the development of the testis (Fig. 1a). Consistent with this finding, the mRNA levels of TFEB, measured by qRT-PCR, in the developing testis also increased during testis development, with a pronounced increase in expression during 7 dpp to 14 dpp (Fig. 1b), which corresponds to the spermatogonial differentiation and the first instances of meiosis in neonatal testes.

**Fig. 2:**
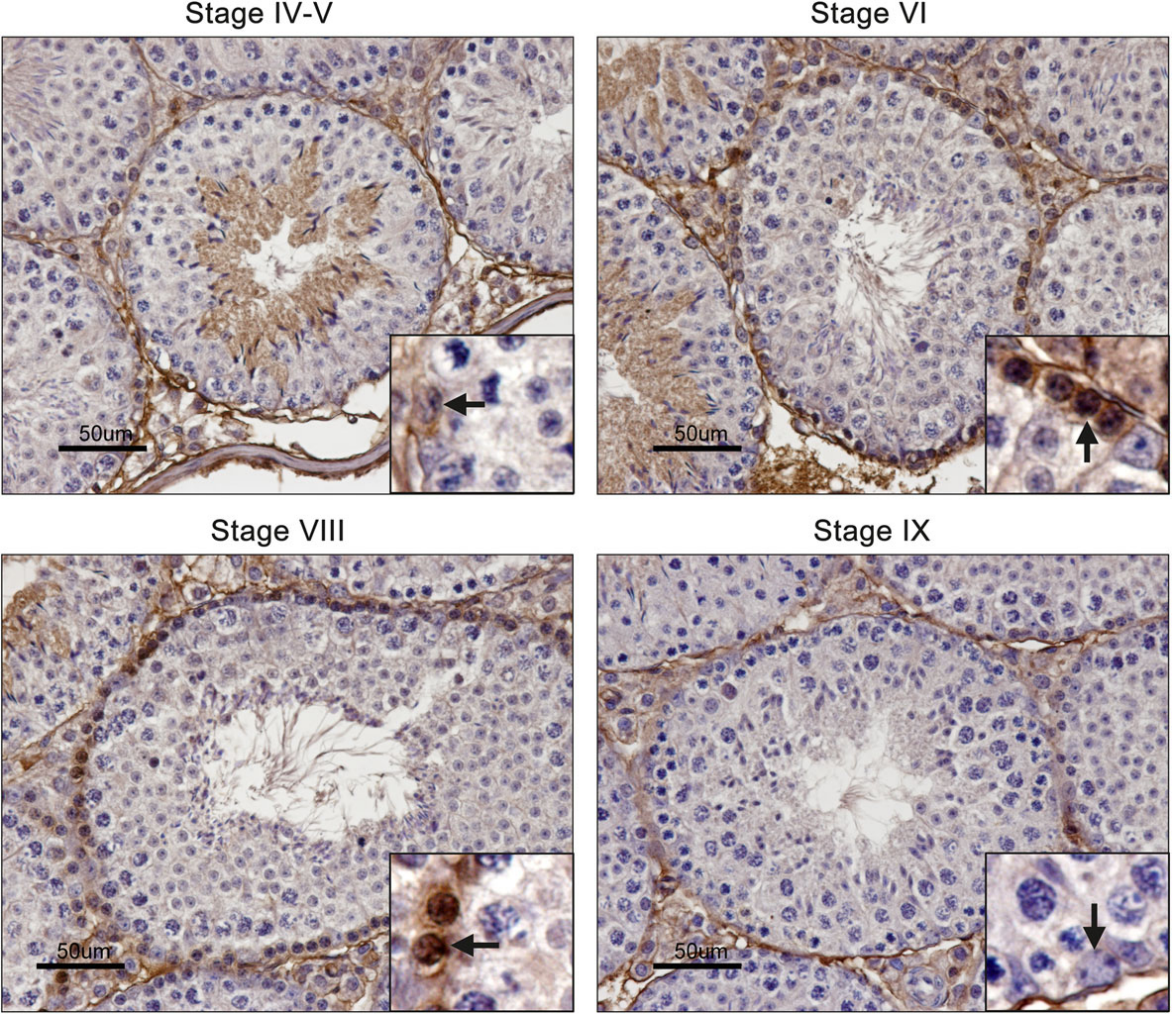
Localization of TFEB in the seminiferous tubules analyzed by immunohistochemistry staining. TFEB significantly expressed in the basal region of seminiferous tubules between stages VI and VIII of the seminiferous epithelial cycle, and was predominantly localized in the nucleus of type B spermatogonia or preL at stages VI and VIII. The type B spermatogonia displayed an oval-shaped nucleus with some heterochromatin clumps, and the preL also displayed chromatin clumps in nucleus. Arrows indicate the spermatogonia in the basal region of seminiferous tubules. Bar: 50 μm

Immunohistochemistry analysis showed that TFEB visibly appeared in spermatogenetic cells in testis after 14 dpp and was mainly expressed in the nucleus of spermatogonia located along the basal region of the seminiferous epithelium as well as in testicular interstitial cells (Fig. 1c). According to the morphological analysis of the seminiferous tubules in adult mice, the immunohistochemistry staining of TFEB in spermatogonia or spermatocytes was mainly present in the seminiferous tubules at stage VI to VIII. At stage VI, TFEB was mainly localized in the nucleus of the differentiating spermatogonia, type B spermatogonia, which displayed an oval-shaped nucleus with more heterochromatin, and were close to or in mitosis into preL. Thus, at stage VII and VIII, TFEB was mainly localized in the first appeared spermatocytes, the preL (Fig. 2).

### TFEB was high expressed in differentiating spermatogonia

**Fig. 3:**
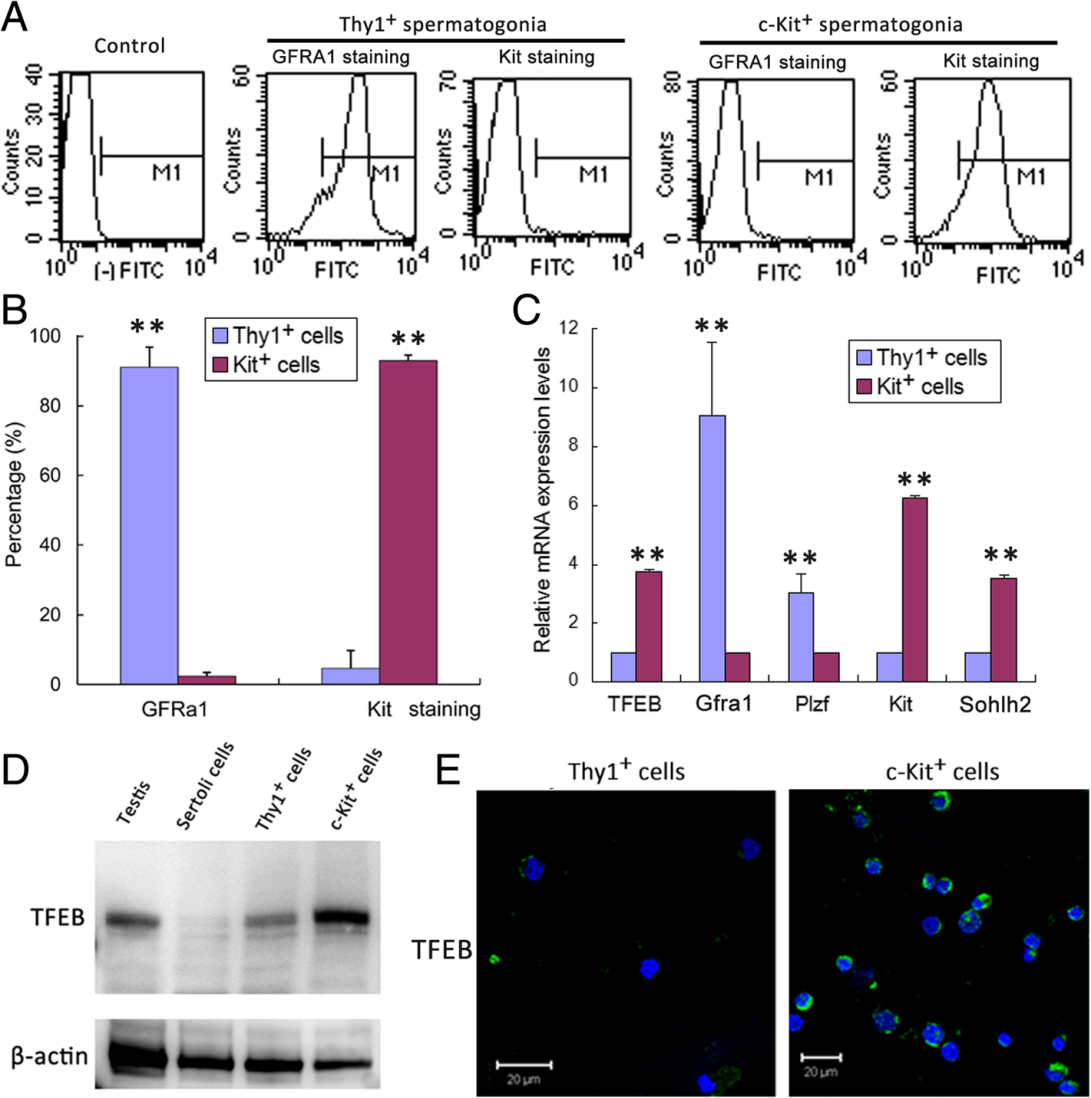
Identification of spermatogonia isolated from 7-day testes by magnetic-activated cell sorting. **a**, **b** The purity of the isolated spermatogonia were identified by flow cytometry analysis, showed that spermatogonia with high purity were isolated by magnetic antibodies. Error bars represent SD (*n* = 5). **p < 0.01. **c** Identification of the isolated spermatogonia by quantitative PCR, showed high levels of *Plzf* and *Gfra1* in Thy1 positive cells and high levels of *TFEB*, *Kit* and *Sohlh2* in c-Kit positive cells. Error bars represent SD (*n* = 5). **p < 0.01. **d** Western blot detection of TFEB in testis, Sertoli cells and isolated spermatogonia, indicated it was mainly expressed in c-Kit positive spermatogonia. **e** Immunofluorescence analysis showed the high expression of TFEB (green fluorescence) in c-Kit positive spermatogonia. Bar: 20 μm

To confirm the expression of TFEB in spermatogonia, both undifferentiated spermatogonia and differentiating spermatogonia were isolated from the 7 dpp testes using magnetic-activated cell sorting. Anti-CD90.2 (Thy1, a cell surface marker for undifferentiated spermatogonia) was applied to isolate the undifferentiated spermatogonia and anti-CD117 (c-Kit, a cell surface marker for differentiating spermatogonia, spermatocyte and spermatid) was applied to isolate the differentiating spermatogonia in 7 dpp testes. The purity of the isolated cells was 87 ± 6% and 94 ± 3%, respectively, verified by flow cytometry analysis (Fig. 3a, b). Cell type-specific marker gene transcripts of the undifferentiated spermatogonia (i.e. *Plzf* and *Gfra1*) and differentiating spermatogonia (i.e. *Kit* and *Sohlh2*) were identified by qRT-PCR (Fig. 3c). These results indicated that undifferentiated or differentiating spermatogonia were successfully isolated with high purity using magnetic-activated cell sorting.

Using qRT-PCR, we found that *TFEB* mRNA was relatively abundant in the c-Kit positive, differentiating spermatogonia (Fig. 3c). Immunoblotting and immunofluorescence analysis also confirmed high levels of TFEB protein in c-Kit positive, differentiating spermatogonia (Fig. 3d, e).

### Primary culture of undifferentiated spermatogonia and induced spermatogonia differentiation by retinoic acid (RA) treatment

**Fig. 4:**
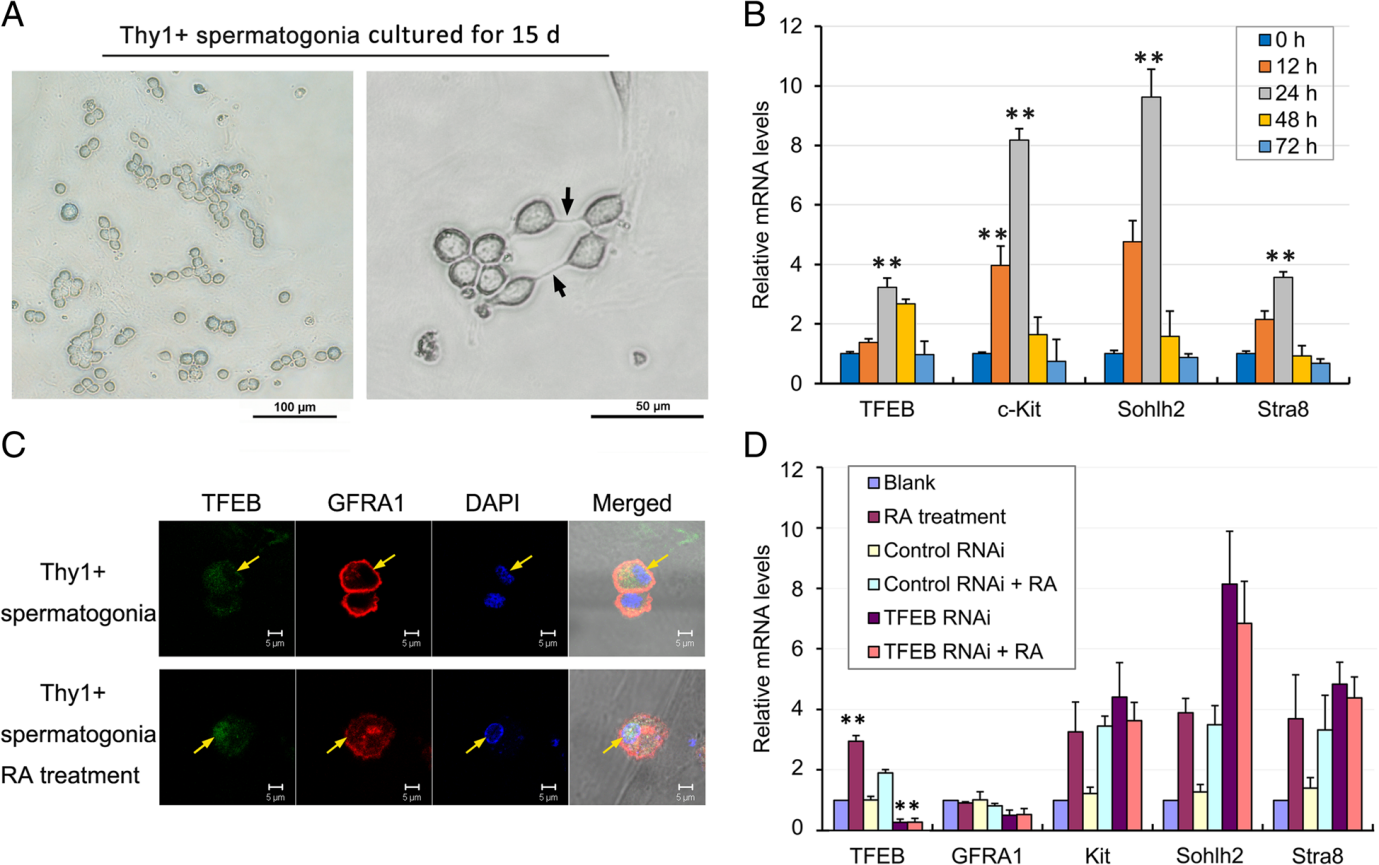
Culture of isolated Thy1 positive spermatogonia and treatment with retinoic acid (RA). **a** The cell morphology of Thy1 positive spermatogonia cultured for 15 days, showed single, paired and aligned cells. Arrows indicate the intercellular bridges. Bar: 100 μm and 50 μm. **b** The mRNA levels of *TFEB* and spermatogonial differentiation markers, *Kit*, *Sohlh2* and *Stra8*, in cultured spermatogonia with RA treatment, showed that expression levels of TFEB and spermatogonial differentiation markers were maximized after RA treatment for 24 h. Error bars represent SD (*n* = 6). **p < 0.01. **c** Immunofluorescence detection of TFEB (green fluorescence) and GFRA1 (red fluorescence) in cultured spermatogonia showed greatly reduced GFRA1 in cell surface and nuclear translocation of TFEB after RA treatment. Arrows indicated the nucleus. Bar: 5 μm. **d** The mRNA levels of *TFEB* and spermatogonial markers in cultured spermatogonia with RNAi and RA treatment. TFEB expression was significantly increased after RA treatment for 24 h, Error bars represent SD (*n* = 6). **p < 0.01. and significantly decreased after shRNA lentiviral transduction. Error bars represent SD (*n* = 6). * *p* < 0.05

To simulate spermatogonia differentiation in vitro, the purified Thy1 positive spermatogonia were cultured and then treated with RA to induce cell differentiation. Freshly isolated, Thy1 positive spermatogonia were cultured on laminin coated dishes and consisted of single, paired and aligned cells after being cultured up to 15 days (Fig. 4a). As shown, paired or aligned cells were connected to each other by intercellular bridges (Fig. 4a). Moreover, the cultured cells were identified as undifferentiated spermatogonia by immunofluorescent staining of cell marker, GDNF family receptor alpha 1 (GFRA1) (Additional file 1: Figure S1).

After treatment with RA for 0, 12, 24, 48 and 72 h, the mRNA levels of cell type-specific marker gene transcripts were assessed by qRT-PCR and the results showed that cell markers of the differentiating spermatogonia (i.e. *Kit*, *Sohlh2* and *Stra8*) were highly expressed after RA treatment for 24 h (Fig. 4b). Meanwhile, immunofluorescence analysis of GFRA1, a marker of undifferentiated spermatogonia, showed that GFRA1 was localized on the membrane of Thy1 positive spermatogonia and was greatly diminished after RA treatment (Fig. 4c). Cumulatively, these results suggested that Thy1 positive spermatogonia were successfully induced to differentiate with RA treatment.

### TFEB activation in response to RA signaling

To investigate the relationship between spermatogonia differentiation and TFEB activation, the purified Thy1 positive spermatogonia were cultured and then treated with RA to induce cell differentiation. In cultured Thy1 positive spermatogonia, the mRNA level of *TFEB* was increased about 3-fold after RA treatment for 24 h (Fig. 4b). More importantly, the ability of TFEB to promote gene transcription is dependent on its nuclear localization, therefore nuclear localization is a marker for the transcription activity of TFEB. Immunofluorescence analysis showed that TFEB localized in the cytoplasm of Thy1 positive, undifferentiated spermatogonia, while it translocated into the nucleus following RA treatment (Fig. 4c). These results suggested that TFEB was activated by RA signaling associated with spermatogonia differentiation.

### Reduced expression of TFEB by RNAi did not interfere spermatogonia differentiation induced by RA

To test whether TFEB was required for RA induced differentiation of spermatagonia, short hairpin RNA (shRNA) expressed by lentiviral vectors (pLenti X1 Puro-shRNA-eGFP-1) were used to knockdown the expression of TFEB. 72 h after shRNA lentiviral transduction, *TFEB* mRNA levels were significantly decreased, by about 73% compared to untreated cells (blank) or lentiviral vector transducted cells (control). The knockdown efficiency was not reversed after RA treatment (*n* = 6, Fig. 4d). Moreover, knockdown of TFEB resulted in significantly increased mRNA levels of spermatogonia differentiation markers (i.e. *Kit*, *Sohlh2* and *Stra8*), which were similar to those blank or control cells treated with RA (Fig. 4d). Therefore, these results suggest that TFEB does not directly regulate spermatogonia differentiation but might participate in further spermatogenesis through promoting lysosome biogenesis or endocytosis as it does in somatic cells.

### Reduced expression of TFEB in GC-1 cells interfere cell migration

Notably, the morphological analysis of TFEB localization, which showed TFEB is highly expressed in type B spermatogonia in the seminiferous tubules from stage VI to VIII, indicated that TFEB might be involved in the preL migrating across the BTB in the seminiferous tubules at stage VIII.

**Fig. 5:**
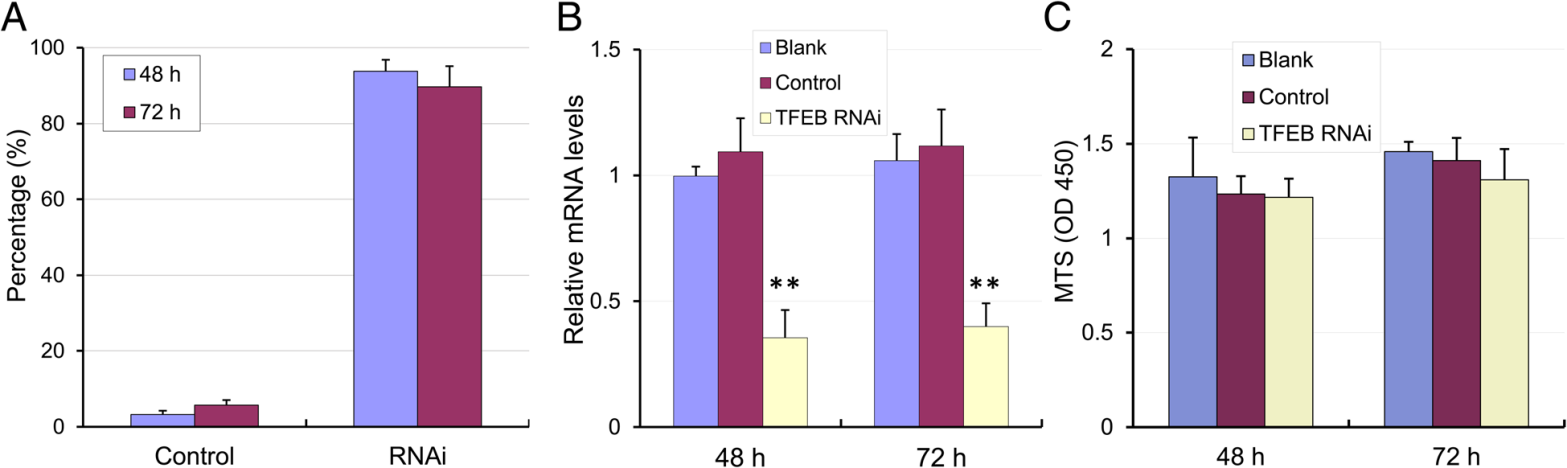
Knock down of TFEB by shRNA lentiviral transduction in GC-1 cells. **a** The lentiviral infection efficiency was accessed by detection of the EGFP using flow cytometer analysis. After lentiviral transduction for 48 h and 72 h, shRNA lentiviral expression efficiency estimated at 94 ± 3% and 90 ± 6%, respectively (*n* = 4). **b** The relative mRNA levels of TFEB after lentiviral transduction for 48 h and 72 h were 0.35 ± 0.11 and 0.40 ± 0.09, comparing to the controls (*n* = 6, ** *p* < 0.01). **c** Cell survival assay performed with MTS method, showed no significant difference after shRNA lentiviral infection for 48 h and 72 h (*n* = 6)

To identify the effect of TFEB on cell migration, both transwell migration assays and wound healing assays were applied to GC-1 cells, a cell line of immortalized spermatogonia. The lentiviral infection efficiency of GC-1 cells was determined with using an EGFP fluorescent marker by flow cytometer analysis. 48 h and 72 h after infection, the EGFP positive cell rate was estimated at 94% and 9%, respectively (*n* = 4, Fig. 5a). TFEB mRNA levels were significantly knocked down at 48 h and 72 h after infection, as compared to mock-infected controls and the efficiency of TFEB knockdown was 65% and 60%, respectively (*n* = 6, Fig. 5b). Moreover, cell survival assays, performed with the MTS method, indicated that knockdown of TFEB had no significant effect on cell viability at 48 h and 72 h after infection (*n* = 6, Fig. 5c), suggesting that knock down of TFEB in GC-1 cells was appropriate for further cell migration experiments between 48 h and 72 h after lentiviral infection.

**Fig. 6:**
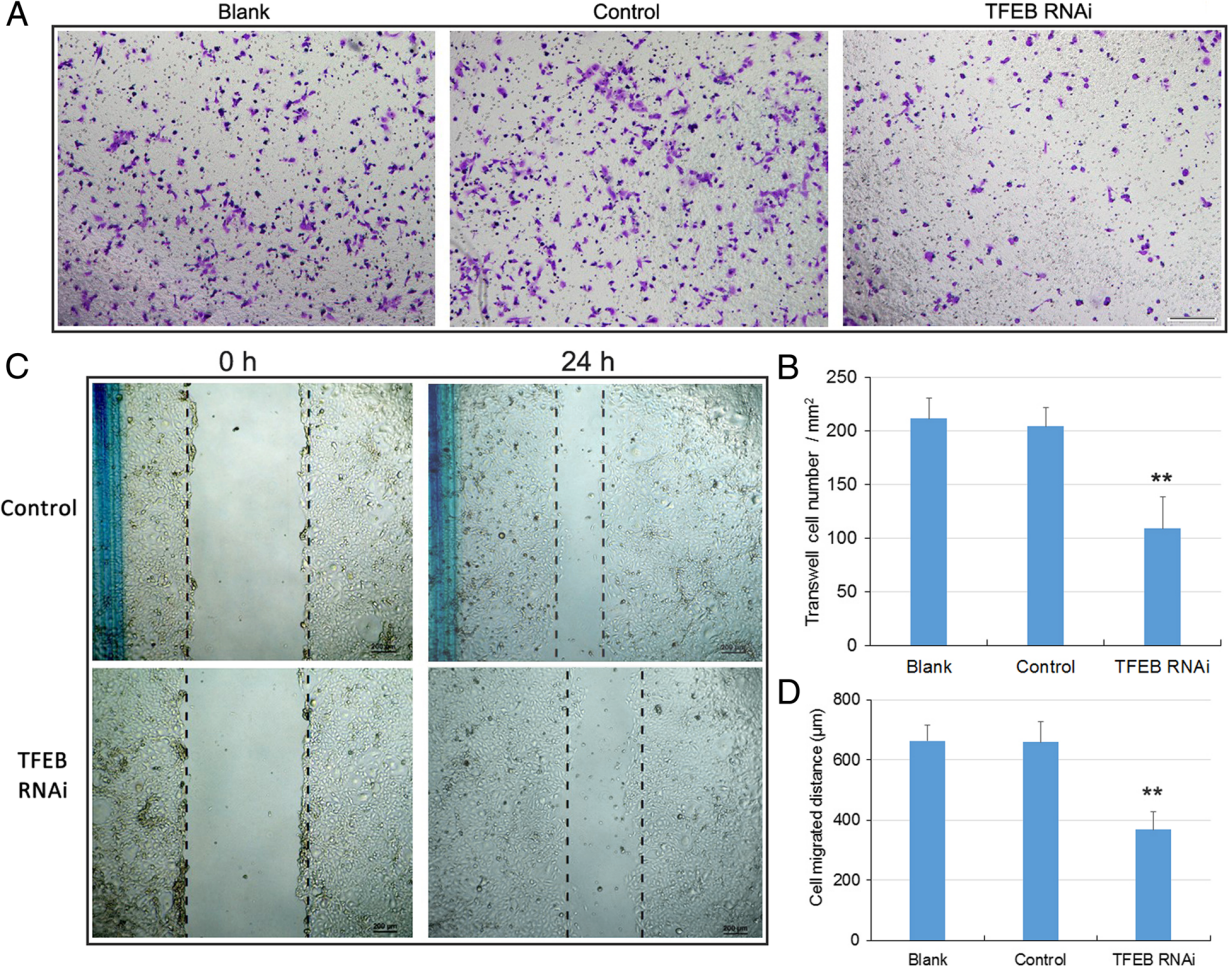
Cell migration assays in GC-1 cells. **a** Representative photographs of the transwell assay show the cells that penetrated through the transwell. Bar: 200 μm. **b** Statistical analysis of transwell assay, showed significant lower migrated cells in TFEB knockdown group than that in blank or control groups (*n* = 5, ** *p* < 0.01). **c** Representative photographs of wound healing assay, showed the cell migrating distance. Bar: 200 μm. (D) Statistical analysis of wound healing assay, showed migrating distance in TFEB knockdown group was significant lower than that in blank or control groups (*n* = 5, ** *p* < 0.01)

In transwell assay, after incubation for 24 h, we counted the number of cells that had penetrated through the transwell (Fig. 6a). The number of migrated TFEB knockdown cells (109 ± 30 / mm^2^) was significantly lower than that of the blank or control cells (Blank, 211 ± 19 / mm^2^; Control, 204 ± 17 / mm^2^. *n* = 5, *P* < 0.01. Fig. 6b), suggesting that the migration ability in TFEB knockdown group was greatly suppressed. In the wound healing assay, knockdown of TFEB resulted in slower cell migration after 24 h of incubation (Fig. 6c), as the distance of migration was significantly smaller in cells with TFEB knock down compared with those in blank and control (Blank, 664 ± 51 μm; Control, 659 ± 68 μm; TFEB RNAi, 368 ± 61 μm. *n* = 5, *P* < 0.01. Fig. 6d). Cumulatively, these results indicated that the down-regulation of TFEB exerted a repressive effect on cell migration.

## Discussion

The present study has clearly demonstrated the expression of TFEB in mouse testicular development, especially ranging from day 14 to the adult stage. Further we show the specific localization of TFEB in the nucleus of the type B spermatogonia and preL in stage VI to VIII of mouse testis. Expression of TFEB was observed in isolated c-Kit positive, differentiating spermatogonia and its nuclear translocation was associated with cell differentiation induced by RA signaling. Furthermore, knockdown of TFEB in immortalized spermatogonia (GC-1 cells) can inhibit cell migration. These observations, together with the reported master regulating effect of TFEB on lysosome function and autophagy, suggest its important role in germ cell migration.

TFEB has been characterized as a master regulator of lysosomal biogenesis and autophagy that promotes the intracellular clearance or recycling of cellular components. Notably, autophagy and endocytosis can efficiently transport sets of transcription factors, adhesion molecules, or secreted factors, and therefore has been recently recognized as a major mechanism in the regulation of self-renewal and differentiation of stem cells as well as a regulator of cell migration [37–39]. Meanwhile, the maintenance of sustained spermatogenesis relies on the balance between spermatogonia self-renewal and differentiation. Previous studies have shown that the mechanistic target of rapamycin complex 1 (mTORC1) is the central regulator of this balance, while it also negatively regulated TFEB activity and restricted the induction of autophagy [40, 41]. These results prompted us to evaluate the relationship between TFEB expression and spermatogenesis, during which several important processes, i.e. spermatogonia differentiation, germ cell migration, acrosome biogenesis and spermiation, seem related to lysosomal function and autophagy.

Interestingly, in mouse testicular development, the expression of TFEB was rapidly increased in the period from 7 dpp to 14 dpp, during which spermatogonia underwent differentiation induced by RA in order to enter the prophase of the first meiotic division. Meanwhile, in the adult testis, the distribution of TFEB appears to be focused in the differentiating spermatogonia at stages VI to VIII of the seminiferous epithelial cycle, which coincides with the germ cell stages that are exposed to the highest levels of RA that promote spermatogonia differentiation, meiotic initiation and preL migrating across the BTB [6, 7]. This seems to indicate that TFEB exhibits a potential association with RA signaling and may be involved in the differentiation of spermatogonia and the release of differentiating germ cells from the basal lamina to migrate across the seminiferous epithelium.

However, the expression pattern of TFEB in testis was insufficient to access the role of TFEB in spermatogonia. It is necessary to purify the spermatogonia and study the TFEB in spermatogonia in vitro. Notably, in 7 dpp testes, spermatogonia are undergoing differentiation but do not initiate meiosis. Thus, both undifferentiated and differentiating spermatogonia account for a large proportion in 7 dpp testes, which is propitious to the isolation of spermatogonia without hindrance from excess spermatocyte and spermatid. Therefore, both undifferentiated and differentiating spermatogonia were isolated from 7 dpp testes successfully by using magnetic-activated cell sorting and the isolated undifferentiated spermatogonia were further cultured with a feeder cell free method. Cell morphology, monitored for over 15 days, showed a unique single, paired and aligned cell arrangement, analogous to the A-single, A-paired and A-aligned spermatogonia in the testis. More importantly, these cultured, undifferentiated spermatogonia could undergo differentiation induced by RA treatment. Our analyses suggest an important model wherein the highly pure populations of spermatogonia can be isolated by magnetic activated cell sorting and used to generate enriched cultures of mouse undifferentiated spermatogonia or spermatogonial stem cells for use in in vitro culture studies on spermatogonial differentiation and meiotic initiation. Based on this method, we found that TFEB was mainly expressed in differentiating spermatogonia and potentially activated by RA signaling, consistent with its expression pattern in testicular sections. As previously described, RA is a novel inducer of spermatogonia differentiation and meiotic initiation [6, 7]. RA associated with retinoic acid receptors (RAR) activates the transcription of *Stimulated by retinoic acid gene 8* (*Stra8*) and *Rec8*, as well as the kinase activity of PI3K/PDK1/AKT/mTORC1 signaling pathways, which are required for translation of *Kit, Sohlh1,* and *Sohlh2,* and promote spermatogonial differentiation [42, 43]. Therefore, we hypothesis that RA signaling might either promote TFEB transcription or regulate TFEB activity through kinase signaling pathways. The association of TFEB activation with RA signaling further highlights a potential role for TFEB in the regulation of certain process during spermatogonial differentiation and meiotic initiation.

Yet, less is known on the mechanisms regulating spermatogonial differentiation [44]. The processes leading from spermatogonial stem cells (SSC) to type B spermatogonia and preL have been seldom investigated. Thus, it is important to better understand the molecular mechanisms involving in these processes. Our data provides a potential candidate responsible for spermatogonial differentiation induces by RA. Thus, this evidence prompted our research to focus on the TFEB function in spermatogonial differentiation. Until now, the biological relevance of TFEB to spermatogonia function or spermatogenesis has largely remained unexplored. Here, we attempted to determine the function of TFEB by RNA interference in cultured spermatogonia. Despite the reduction in TFEB mRNA expression levels, the anticipated decline in the levels of spermatogonial differentiating markers was not observed, which suggests other possible function of TFEB than directly induce spermatogonia differentiation should be taken into consideration.

Therefore, considering that TFEB is a master regulator of lysosomal biogenesis and autophagy, we hypothesized that TFEB expression in differentiating spermatogonia may promote cell migration that is required for crossing the BTB. Recent studies have found that knockdown of TFEB by siRNA transfection has no effects on proliferation but diminishes migration abilities in several cancer cell lines [28, 29]. Here, our study provides the first evidence for the role of TFEB in spermatogonial migration. Primary cultured spermatogonia were inadaptable to the classical cell migration assays because of the low cellular density and poor cell adhesion. In a previous study, Chen et al. performed a migration assay in immortalized spermatogonia, GC-1 cells [36]. Although it might lose some characteristics of normal spermatogonia, GC-1 cells are the most compatible cell line representing spermatogonia and are applied in many experiments to evaluate spermatogonia function in vitro [45–47]. Moreover, TFEB expressed in GC-1 cells also exhibited nucleus translocation following RA treatment (Additional file 1: Figure S3), which was consistent with that in primary cultured spermatogonia. This result indicated to the extent that GC-1 cells were appropriate for studying the effect of TFEB. Thus, in present study, GC-1 cells were also applied in cell migration experiments using the transwell and wound healing methods. The results confirmed that knockdown of TFEB led to an impairment in cell migration and indicated the potential effect of TFEB on spermatogonia migration, which are important and guided for the further study. Although the mechanisms by which the TFEB impacts migration is currently unknown, one possible explanation is that TFEB is involved in the degradation of matrix proteins, adhesion proteins and chemotactic receptors by activation of endocytosis, autophagy and lysosomal degradation.

On the other hand, the underlying mechanism for regulating migration of spermatogonia or spermatocytes is mostly unknown at present. Previous studies on germ cell transport or migration across the seminiferous epithelium, most notably the transport of preL across the BTB and the transport of elongating spermatids across the adluminal compartment, are focused on the adhesion protein complexes at the cell-cell interface [48–50]. It is generally accepted that cell junctions elaborated by Sertoli cells, namely both Sertoli-Sertoli cell and Sertoli-germ cell junctions, contribute to germ cells migration across the seminiferous epithelium. Emerging evidence has shown that junction dynamics at the Sertoli-Sertoli cell and Sertoli-germ cell interface are supported by the intriguingly coordinated cytoskeletons, microfilament- and microtubule-based cytoskeletons. However, apart from the effect of Sertoli cells, the molecular mechanisms in germ cell itself for its timely and efficient migration across the BTB remain virtually unknown. Limited evidences of the mechanisms in germ cell migration were found in studies on the homing or migration of pro-spermatogonia into the SSC niche. Earlier researches suggested that small G protein Rac1 and transcription factor RHOX10 are critical regulators of spermatogonial stem cells (SSC) transmigrate through the BTB and migrate into niches during SSC homing [51, 52]. Herein, our results indicate that TFEB as a transcriptional factor in spermatogonia has potential function in promoting spermatogonia migration, therefore provide a new candidate responsible for spermatogonia migration.

Whereas additional studies are required to understand precisely how stage-specific expression and activation of TFEB are regulated by RA signaling and how its downstream target genes impact on spermatogenesis, our immediate goal was to set the stage for assessing the role of TFEB in spermatogonia.

## Conclusions

In conclusion, we demonstrated, for the first time, that TFEB expressed in spermatogonia in mouse testis and its expression was associated with spermatogonial differentiation and meiotic initiation. These data suggest that regionally distinct expression and activation of TFEB was strongly associated with RA signaling, and therefore may contribute to promote cell migration across the BTB and transport along the seminiferous epithelium.

## Additional file

Additional file 1: **Figure S1.** Isolation and culture of spermatogonia from 7-day testes. (a-b) Enzymatic digestion of seminiferous tubules into fragments and single cells. (c) Testicular single cells were further performed differential attachment, showed the somatic cells present as triangle- or spindle-liked forms were attached to the plate, whereas the round unattached cells were spermatogonia. (d) Spermatogonia purified by magnetic-activated cell sorting. (e) Culture of the somatic cells isolated by differential attachment, containing Sertoli cells and myoid cells. (f) Culture of the Thy1 positive spermatogonia. Bar: 200 μm in (a), (b); 100 μm in (c)-(e); 50 μm in (f). **Figure S2.** Immunofluorescent staining of GFRA1 in cultured spermatogonia. The result showed most cells were positive for GFRA1, suggesting high purity of undifferentiated spermatogonia. Bars: 20 μm. **Figure S3.** Immunofluorescent staining of TFEB in GC-1 cells. The result showed that RA treatment induced TFEB nucleus translocation. Bars: 20 μm. **Table S1.** Oligonucleotide primer sequences used for qRT-PCR. (DOCX 3401 kb)

## Abbreviations

ANOVA: One-way analysis of variance
BSA: Bovine serum albumin
BTB: Blood-testis barrier
dpp: Days post-partum
EGFP: Enhanced green fluorescence protein
FBS: Fetal bovine serum
GFRA1: GDNF family receptor alpha 1
H&E: Hematoxylin and eosin
IHC: Immunohistochemistry
mTORC1: Mechanistic target of rapamycin complex 1
preL: Preleptotene spermatocytes
PVDF: Polyvinylidene difluoride
RA: Retinoic acid
RT-PCR: Reverse transcription-polymerase chain reaction
shRNA: Short hairpin RNA
TFEB: Transcriptional factor EB
