# Supplementary material for: Expression of transcriptional factor EB (TFEB) in differentiating spermatogonia potentially promotes cell migration in mouse seminiferous epithelium
Source: Reprod Biol Endocrinol. 2018 Oct 25;16:105. doi: 10.1186/s12958-018-0427-x (PMC6202848; doi:10.1186/s12958-018-0427-x)
Supplement: Supplementary file 1 — Figure S1. Isolation and culture of spermatogonia from 7-day testes. (a-b) Enzymatic digestion of seminiferous tubules into fragments and single cells. (c) Testicular single cells were further performed differential attachment, showed the somatic cells present as triangle- or spindle-liked forms were attached to the plate, whereas the round unattached cells were spermatogonia. (d) Spermatogonia purified by magnetic-activated cell sorting. (e) Culture of the somatic cells isolated by differential attachment, containing Sertoli cells and myoid cells. (f) Culture of the Thy1 positive spermatogonia. Bar: 200 μm in (a), (b); 100 μm in (c)-(e); 50 μm in (f). Figure S2. Immunofluorescent staining of GFRA1 in cultured spermatogonia. The result showed most cells were positive for GFRA1, suggesting high purity of undifferentiated spermatogonia. Bars: 20 μm. Figure S3. Immunofluorescent staining of TFEB in GC-1 cells. The result showed that RA treatment induced TFEB nucleus translocation. Bars: 20 μm. Table S1. Oligonucleotide primer sequences used for qRT-PCR. (DOCX 3401 kb) [file 12958_2018_427_MOESM1_ESM.docx]

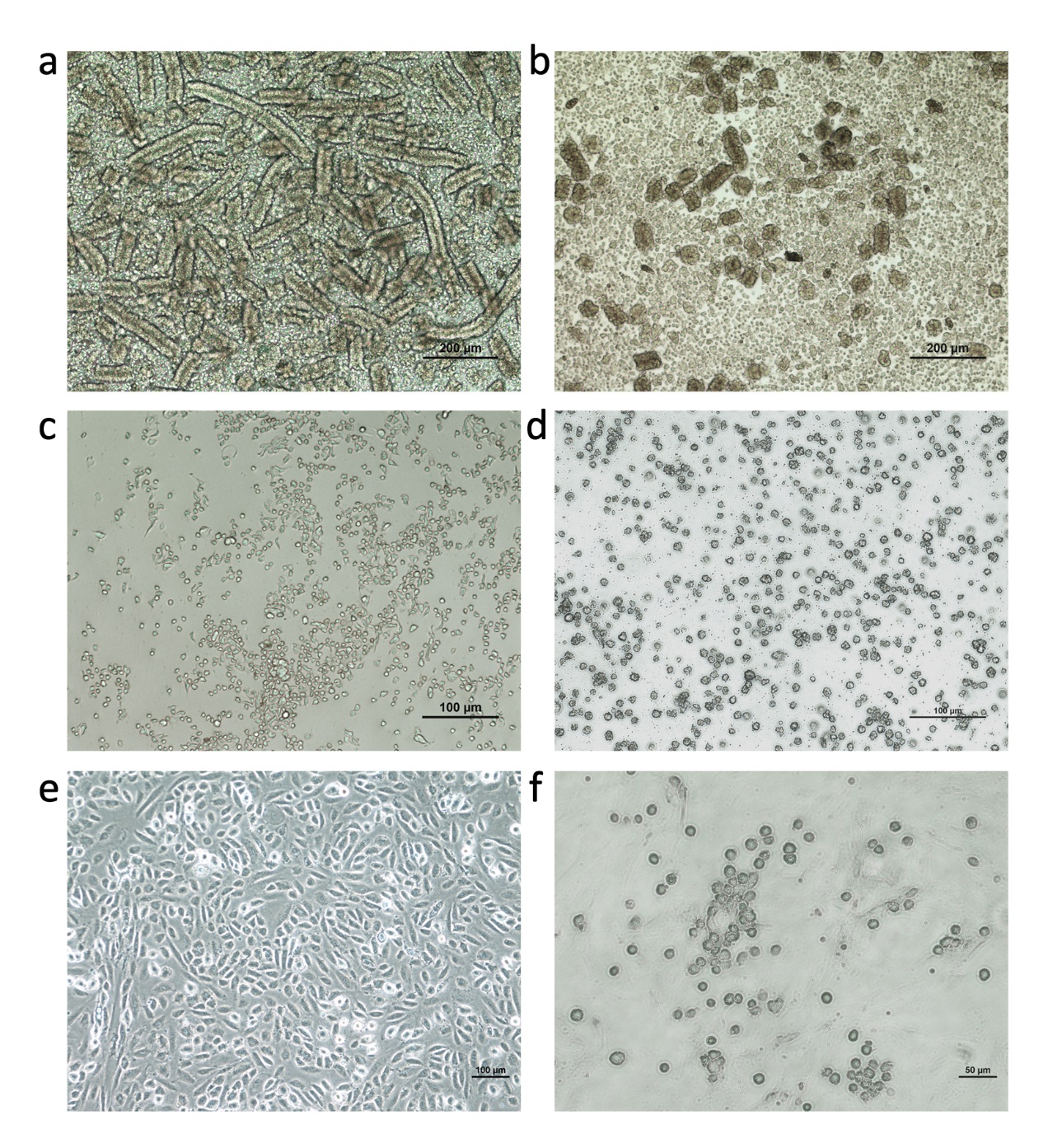
Additional file 1: **Figure S1** Isolation and culture of spermatogonia from 7-day testes. (a-b) Enzymatic digestion of seminiferous tubules into fragments and single cells. (c) Testicular single cells were further performed differential attachment, showed the somatic cells present as triangle- or spindle-liked forms were attached to the plate, whereas the round unattached cells were spermatogonia. (d) Spermatogonia purified by magnetic-activated cell sorting. (e) Culture of the somatic cells isolated by differential attachment, containing Sertoli cells and myoid cells. (f) Culture of the Thy1 positive spermatogonia. Bar: 200 μm in (a), (b); 100 μm in (c)-(e); 50 μm in (f).

**Figure S
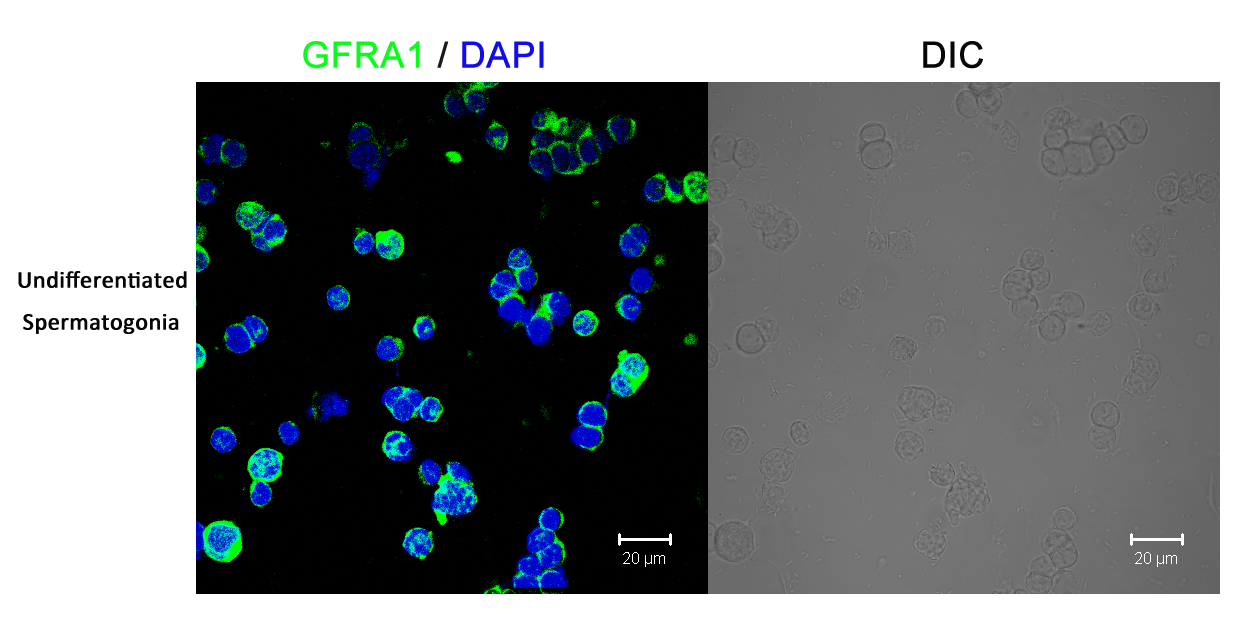
2** Immunofluorescent staining of GFRA1 in cultured spermatogonia. The result showed most cells were positive for GFRA1, suggesting high purity of undifferentiated spermatogonia. Bars: 20 μm.


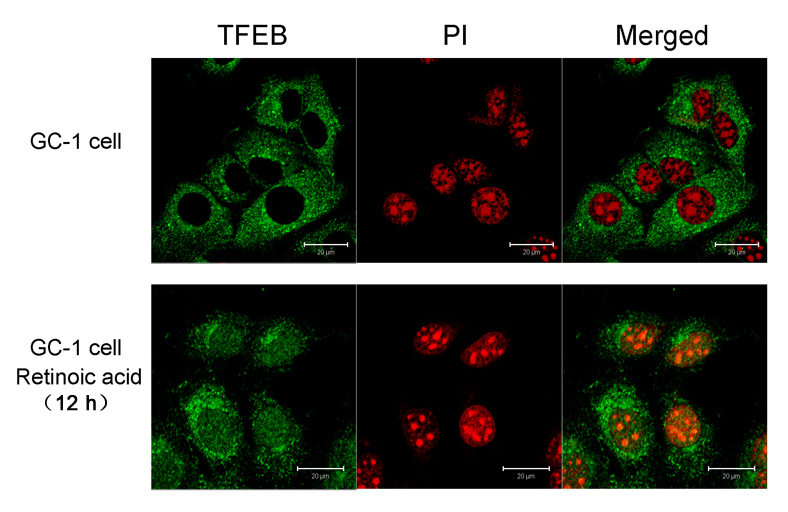


**Figure S3** Immunofluorescent staining of TFEB in GC-1 cells. The result showed that RA treatment induced TFEB nucleus translocation. Bars: 20 μm.

| **Table S1** Oligonucleotide primer sequences used for qRT-PCR | | |
| --- | --- | --- |
| Gene | Forward Primer (5’-3’) | Reverse Primer (5’-3’) |
| *Tfeb* | TCAGAAGCGAGAGCTAACAGAT | TGTGATTGTCTTTCTTCTGCCG |
| *Gfra1* | CTAGCCACTCTGTACTTCGT | GCTTGCAGCGGCAGTTGTAGA |
| *Kit* | GCCACGTCTCAGCCATCTG | GTCGGGATCAATGCACGTCA |
| *Plzf* | ATGGACTTCAGCACCTACGG | TGCATTCTCAGTCGCAAACT |
| *Sohlh2* | GGGCAGGGCAGAGTAAATCTT | CAAACGAGTTAGCAGCCAAAAG |
| *Stra8* | CCTGGTTGAGGGGTGTAAGG | ATCACAGCCCTGTCACTGC |
| *ActB* | CCAGTTCGCCATGGATGACGATAT | GTCAGGATACCTCTCTTGCTCTG |
